# Invasive Sinusitis With Arcanobacterium haemolyticum and Fusobacterium necrophorum Complicated by Subdural Empyema in an Immunocompetent Adolescent Patient

**DOI:** 10.7759/cureus.44517

**Published:** 2023-09-01

**Authors:** Hanna S Sahhar, Erica Rubin, Sami E Rishmawi, Matthew Logan

**Affiliations:** 1 Pediatric Intensive Care Unit, Spartanburg Regional Healthcare System, Spartanburg, USA; 2 Pediatrics, Edward Via College of Osteopathic Medicine, Spartanburg, USA

**Keywords:** sinusitis, cerebral edema, subdural empyema, arcanobacterium haemolyticum, fusobacterium necrophorum

## Abstract

We are reporting a very rare case of an invasive infection with *Arcanobacterium haemolyticum* and *Fusobacterium necrophorum *that resulted in meningitis, cerebral edema, and subdural empyema secondary to upper respiratory infection (URI) and sinusitis in an immunocompetent adolescent patient. Our patient is a 17-year-old male with no significant medical history who presented to his pediatrician with a fever for three days, was diagnosed with a viral URI, and instructed to continue symptomatic care. Seven days later, the patient developed a headache, left-sided weakness, and continued to spike fever. The patient presented to the Emergency Center due to altered mental status, worsening left-sided weakness, and difficulty speaking. Head computed tomography (CT) scan showed small right-sided fluid collection with right-to-left midline shift and marked opacification of paranasal sinuses with air-fluid levels in frontal sinuses. The patient underwent an emergent craniotomy that revealed subdural empyema under high pressure and was started on vancomycin, cefepime, metronidazole, and levetiracetam. Six hours after his craniotomy, the patient developed fixed dilatation of his right-side pupil and a head CT scan showed developing ischemic changes and increased in his midline shift which prompted to emergent right decompressive craniectomy. The following day of his surgery, magnetic resonance imaging of the brain showed large acute infarctions of the right hemisphere, edema, and subfalcine herniation. Two brain death exams - 12 hours apart - were performed in which criteria for brain death were met. The patient’s subdural empyema culture grew *Fusobacterium necrophorum* and *Arcanobacterium haemolyticum*.

## Introduction

*Arcanobacterium haemolyticum* is a gram-positive pleomorphic rod that is a facultative anaerobe and catalase-negative. *A. haemolyticum* infections are responsible for around 2.5% of pharyngitis infections [1]. It is most common in the 15- to 25-year-old age group and responsible for 3% of pharyngeal infections [2]. This pathogen often presents similar to group A *Streptococcal *infections with fever, exudate, erythema, rash, and cervical lymphadenopathy [1]. Invasive infections of *A. haemolyticum* can cause peritonsillar abscess, Lemierre syndrome, bacteremia, sepsis, endocarditis, brain abscess, orbital cellulitis, pyogenic arthritis, or rarely, other infections, though these are typically seen in immunosuppressed patients [1]. *A. haemolyticum* is often seen in polymicrobial infections [2]. It is thought that transmission of the bacteria is from person to person through droplet exposure [2]. Diagnosis of this bacterium is usually only after recurrent infections which may be due to missed diagnosis and improper treatment [2]. *A. haemolyticum* is susceptible to erythromycin or azithromycin and is commonly resistant to trimethoprim-sulfamethoxazole [1].

*Fusobacterium necrophorum* is a gram-negative rod that is anaerobic. It can be found in the oropharynx of healthy people and is a common component of dental plaques. It can also be found in the respiratory tract of many animals, as well as in the soil [3]. Recent studies have linked *F. necrophorum* to persistent pharyngitis [4]. *F. necrophorum* infections are most common in young adults typically presenting as otitis media infections, gingivitis, and tonsillitis [3]. It often is seen with concomitant infections with other microorganisms such as beta-hemolytic streptococcus [5]. Lemierre’s syndrome, meningitis, brain abscess, and cerebral venous sinus thrombosis are serious though uncommon complications from an infection with this pathogen [3,5]. Combination therapy with metronidazole or clindamycin, in addition to a beta-lactam against aerobic oral and respiratory tract pathogens (cefotaxime, ceftriaxone, or cefuroxime), is recommended for patients with invasive infection caused by *Fusobacterium *species [1].

Invasive infections of *A. haemolyticum* and *F. necrophorum* are typically seen in immunosuppressed patients [1,3,5].

This article was previously presented as an abstract and poster at the 2020 VCOM Research Recognition Day - Carolinas Campus on April 17, 2020.

## Case presentation

**Figure 1:**
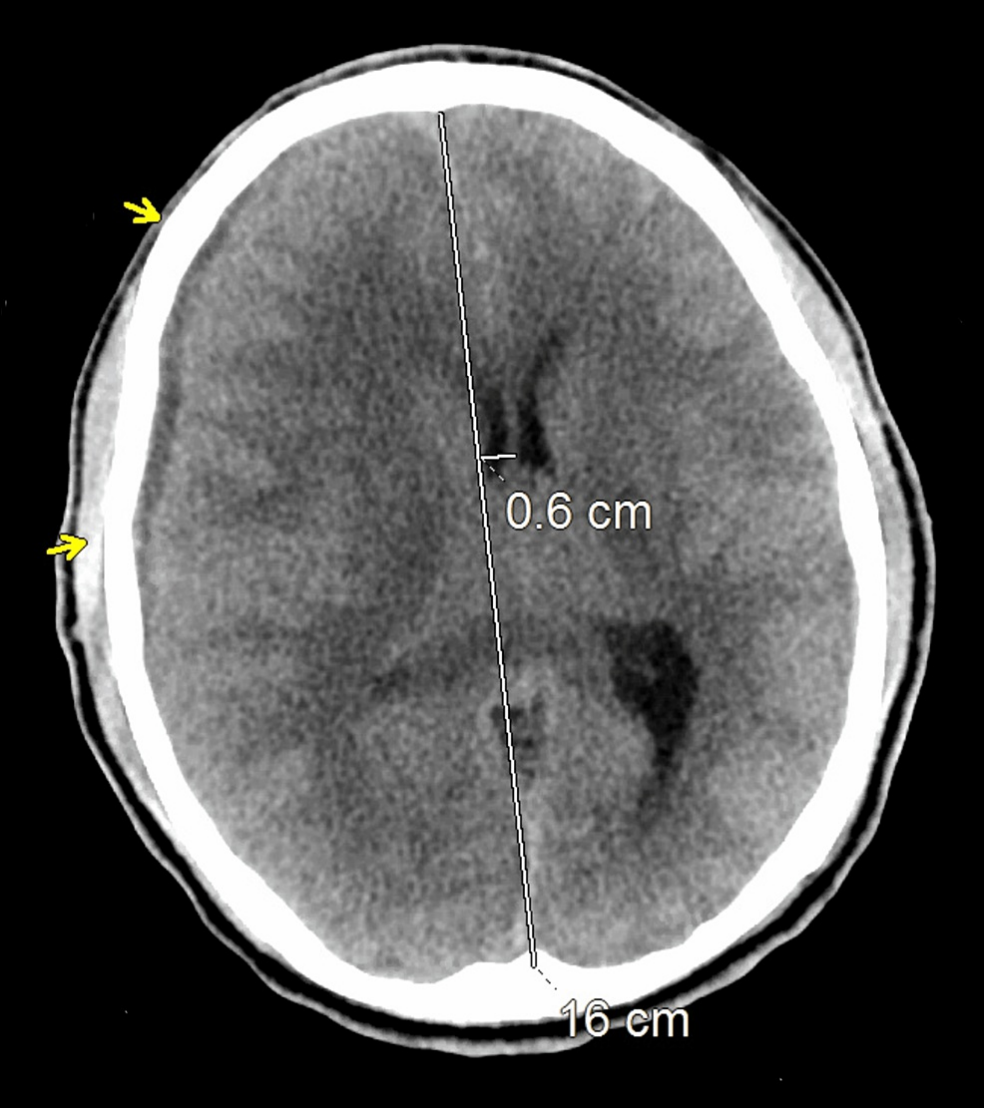
Head CT scan upon presentation to the Emergency Center: Relatively small volume hypodense subdural fluid collection on the right, mass effect with right-to-left shift of midline measured at 6mm with some mild collapse of the right lateral ventricle. CT: computed tomography

**Figure 2:**
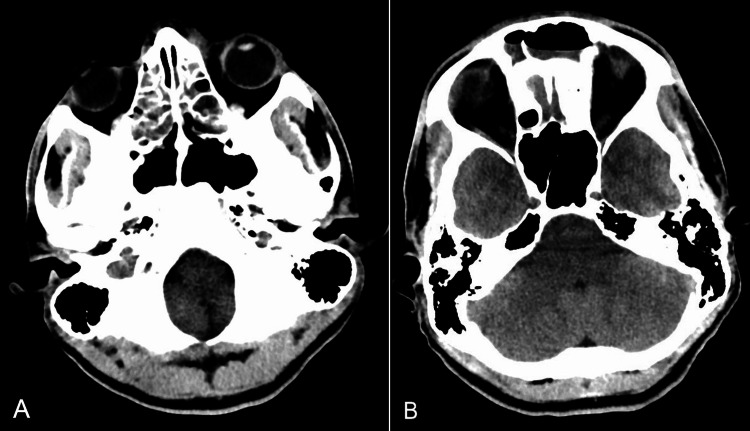
Head CT scan showing marked opacification of paranasal sinuses (A) with air-fluid level in the frontal sinuses (B). CT: computed tomography

A 17-year-old male with no significant medical history presented to his pediatrician with fever, runny nose, cough, and congestion for three days. After negative rapid influenza and streptococcal tests, the patient was diagnosed with an upper respiratory infection (URI) and instructed to continue symptomatic care. Seven days later, the patient developed a headache, facial droop, and left-sided weakness. He continued to have subjective fevers and diaphoresis despite symptomatic treatment. On the day of his admission, he presented to the Emergency Center due to altered mental status, worsening left-sided weakness, and difficulty speaking with a history that he fell at home and hit his head the night before. Physical examination in the Emergency Center showed a confused, diaphoretic, uncomfortable male, with left conjugate gaze paresis and loss of left peripheral vision, left wrist and elbow contracted with no range of motion. The rest of the neurological examination was unremarkable including brain stem function. Initial laboratory studies showed leukocytosis - total white blood cell count 16.1 × 103/uL (normal range 4.5-13.0 × 103/uL), relative neutrophils 97.8%, and hyponatremia with sodium of 132 mmol/L (normal range 133-146 mmol/L). Head computed tomography (CT) scan showed a small right-sided fluid collection with a right-to-left shift of midline and marked opacification of paranasal sinuses with air-fluid levels in frontal sinuses (Figures 1, 2).

**Figure 3:**
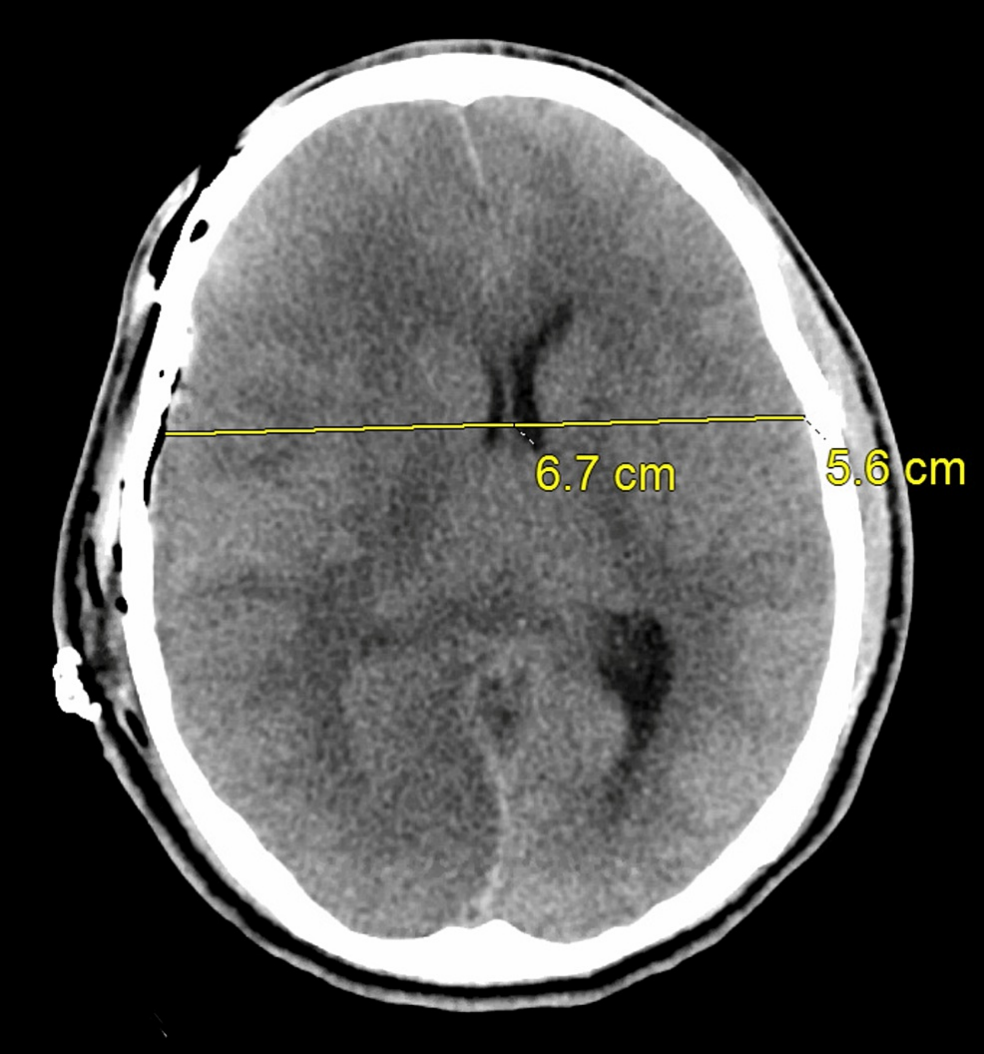
Head CT scan post decompressive craniectomy on day one shows developing ischemic changes involving the anterior right frontal convexity medial right occipital lobe extending into the posterior temporal lobe. CT: computed tomography

The patient underwent an emergent craniotomy that revealed subdural empyema under high pressure; subdural empyema had been removed and the patient was then admitted to the pediatric intensive care unit, intubated, sedated, and mechanically ventilated. The patient was given vancomycin, cefepime, and metronidazole which started before surgery. Levetiracetam and dexamethasone were added a few hours later. The patient required an inotropic support, norepinephrine, due to hemodynamic instability. Bacterial cultures and gram stains of the empyema were taken. Six hours after his craniotomy, he developed fixed dilatation of his right-side pupil, in which a repeated head CT scan showed developing ischemic changes, and an emergent decompressive right frontotemporoparietal craniectomy was performed (Figure 3).

**Figure 4:**
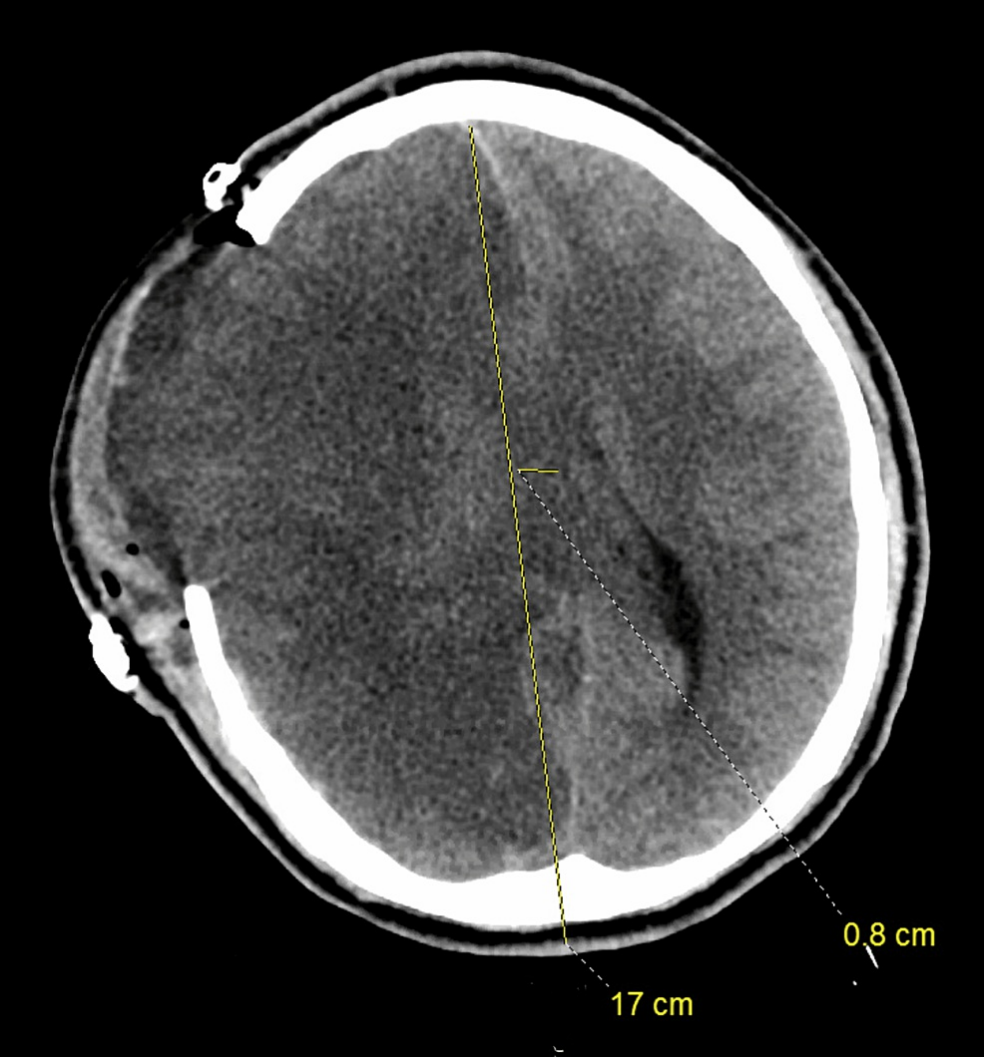
Head CT scan post decompressive craniectomy on day two shows severe cerebral edema in the right hemisphere, mass effect, and approximately 8mm right to left midline shift. CT: computed tomography

Head CT scan on hospital day two showed severe cerebral edema in the right hemisphere, mass effect, and approximately 8 mm right to left midline shift that increased from the previous study (Figure 4).

Laboratory studies on hospital day two showed an increase in total white blood cell count to 26.1 × 103/uL, decrease in hemoglobin to 10.8 g/dL (normal range 13.0-16.0 g/dL), decrease in platelets to 77 × 103/uL (normal range 119-332 × 103/uL), C-reactive protein 29.9 mg/dL (normal range 0.00-0.60 mg/dL), improvement in sodium to 140 mmol/L, decrease in albumin to 2.5 g/dL (normal range 3.1-4.8 g/dL), increasing aspartate aminotransferase to 81 IU/L (normal range 8-36 IU/L), and increase of total bilirubin to 3.3 mg/dL (normal range 0.0-1.5 mg/dL). Magnetic resonance imaging on hospital day three showed extensive areas of large acute infarction of the right hemisphere, edema, and subfalcine herniation. An intracranial pressure monitor was placed to determine further treatment options. Due to elevated intracranial pressure, the decision was made by the family to defer further surgical interventions. On hospital day three, the patient’s first brain death exam was completed and confirmed brain death. Twelve hours later, the second brain death exam was also positive, and the patient was pronounced dead.

**Figure 5:**
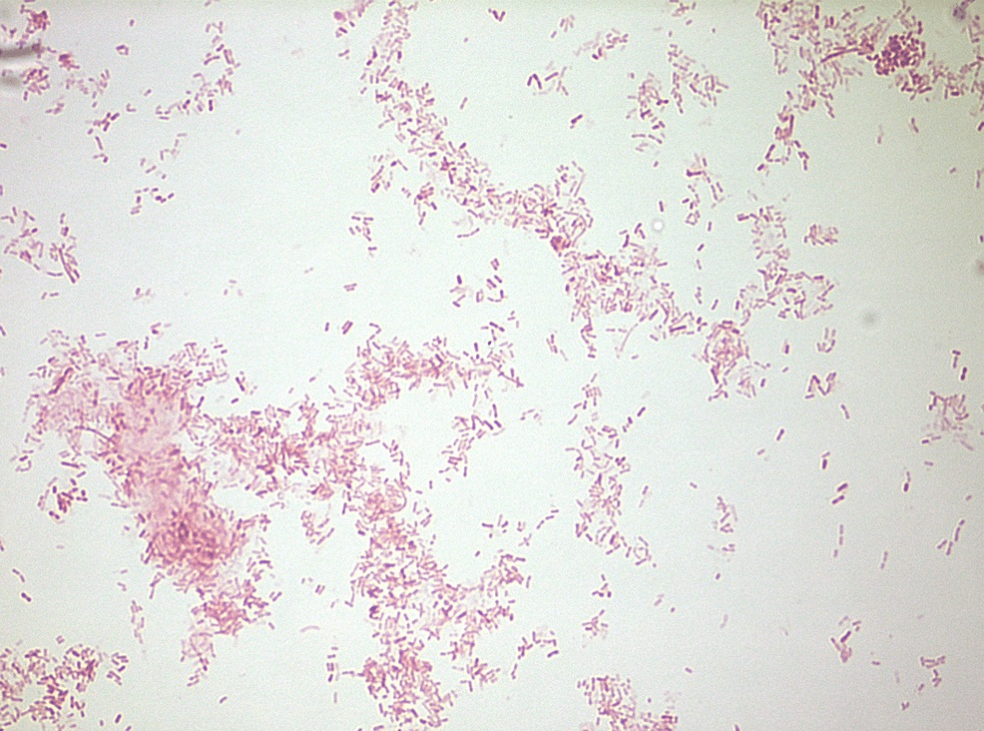
This figure demonstrates the gram-negative bacilli that was identified as Fusobacterium necrophorum.

**Figure 6:**
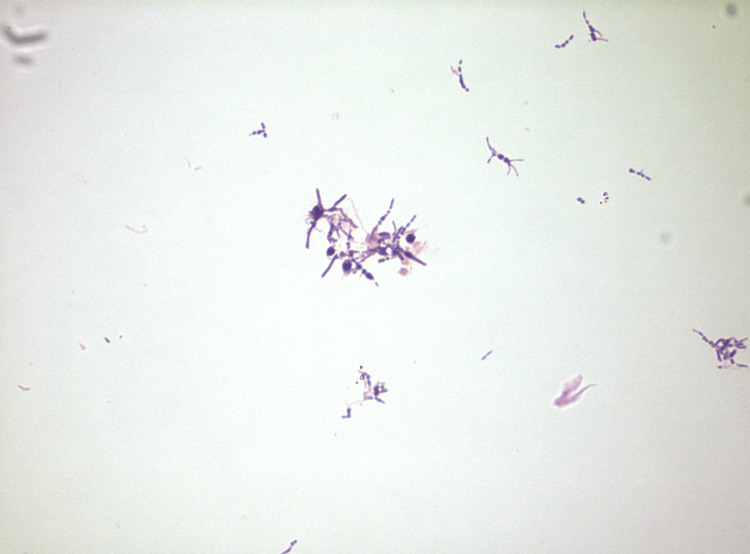
This figure demonstrates the gram-positive bacilli that was identified as Arcanobacterium haemolyticum.

**Figure 7:**
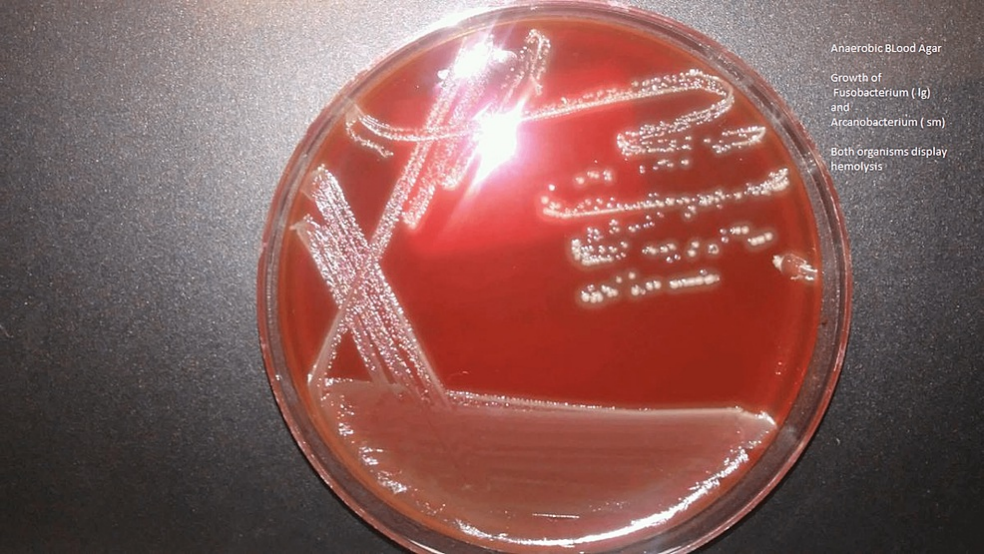
This figure demonstrates the growth and hemolysis of Fusobacterium necrophorum (large colonies) and Arcanobacterium haemolyticum (small colonies) on anaerobic blood agar culture.

The presence of *F. necrophorum* and *A. haemolyticum* was confirmed by gram stain and anaerobic blood agar cultures from the subdural empyema (Figures 5, 6, 7). The sample was collected during the initial craniotomy from the patient’s right subdural empyema. Initial gram stain report showed few polymorphonuclear leukocytes seen, few Gram-negative and Gram-positive bacilli. Bruker MALDI - Identification (Bruker Daltonics, Billerica, MA) was performed on and confirmed the presence of *A. haemolyticum*. Bruker MALDI - Identification of colony growth on the anaerobic culture confirmed the presence of *F. necrophorum* (Figure 7). Susceptibility testing was performed via Kirby Bauer disk diffusion and the pathogens were susceptible to penicillin 0.064 ug/mL, ceftriaxone 31 mm, doxycycline 12 mm, clindamycin 29 mm, rifampin 29 mm, levofloxacin 0.50 ug/mL, linezolid 0.38 ug/mL, and vancomycin 0.50 ug/mL.

## Discussion

As evidenced by the patient’s clinical symptoms and head CT scan, the initial presentation started with upper respiratory symptoms as well as acute sinusitis. The cultures from the subdural empyema revealed the infectious agents are *A. haemolyticum* and *F. necrophorum*. This could represent a contiguous spread from the initial sinus infection and URI. Invasive infections of *A. haemolyticum *and *F. necrophorum* can lead to subdural empyema, brain abscess, meningitis, Lemierre’s syndrome, pneumonia, and bacteremia. In these invasive infections, *A. haemolyticum* is usually a co-pathogen and rarely the sole infectious agent [6]. A subdural empyema is most commonly a complication from acute sinusitis [7]. In some cases, pulmonary arteriovenous malformations must be ruled out as the underlying cause of brain abscesses [8]. The most common agents are anaerobic and microaerophilic *Streptococci*, with other organisms such as *Staphylococcus aureus*, *Escherichia coli*, and *Bacteroides *are less common [7]. While a co-infection of *A. haemolyticum* and *F. necrophorum* resulting in Lemierre’s syndrome has been documented, to our knowledge this is the first case with this co-infection leading to a meningitis with subdural empyema. Complications of this nature are extremely rare, and this case is of interest due to the patient being immunocompetent. The patient also lacked risk factors to increase the opportunity for an invasive *Fusobacterium *infection such as periodontal disease, dental plaques, otitis media, and/or tonsillitis.

These bacteria are susceptible to several different antibiotics. *A. haemolyticum* is susceptible to macrolides, cephalosporins, fluoroquinolones, vancomycin, and tetracyclines [1,6]. *F. necrophorum* is susceptible to metronidazole, clindamycin, carbapenems, chloramphenicol, cefoxitin, and ceftriaxone [3]. Resistances have been increasing so sensitivity testing is recommended as *F. necrophorum* is resistant to penicillin, ampicillin, some cephalosporins, fluoroquinolones, gentamicin, and macrolides [3]. Empiric therapy while sensitivity testing is done should begin with a beta-lactam agent, such as ampicillin-sulbactam, with or without a macrolide, and metronidazole [1]. Due to the patient’s delayed presentation to the Emergency Center, the patient’s first antibiotic exposure was the broad-spectrum antibiotics, vancomycin, cefepime, and metronidazole, initiated after the subdural empyema was discovered in the initial craniotomy.

## Conclusions

While the majority of acute URI/sinusitis are viral and require no antibiotic therapy, physicians and patients need to be aware of when to seek further care. Typically, acute sinusitis/viral URIs are treated with supportive care unless symptoms worsen or persist beyond seven to ten days. Patients need to be aware of what symptoms would indicate a worsening disease course and indicate additional follow-up. Physicians need to communicate and educate on the importance for patients to return when symptoms worsen or persist.

When evaluating a patient for URI, it is important to consider rare organisms and additional follow-up in presumed URI/sinusitis that does not improve within seven days. This case shows the need to quickly identify the possible pathogens to ensure adequate treatment. Currently, there are not any routine or rapid tests for the detection of *F. necrophorum *and *A. haemolyticum*. High clinical suspicion for these pathogens is needed for additional patient testing. Our case highlights the importance of additional patient follow-up and consideration of rare pathogens in acute URI/sinusitis to ultimately prevent serious complications of rare pathogens such as *F. necrophorum* and *A. haemolyticum*.

## References

[1] Kimberlin DW, Brady MT, Jackson MA (2018). Report of the Committee on Infectious Diseases (31st Edition). Brady et al., American Academy of Pediatrics.

[2] Linder R (1997). Rhodococcus equi and Arcanobacterium haemolyticum: two "coryneform" bacteria increasingly recognized as agents of human infection. Emerg Infect Dis.

[3] Kimberlin DW, Barnett ED, Lynfield R, Sawyer MH (2021). Report of the Committee on Infectious Diseases (32nd Edition). Brady et al., American Academy of Pediatrics.

[4] Batty A, Wren MW (2005). Prevalence of Fusobacterium necrophorum and other upper respiratory tract pathogens isolated from throat swabs. Br J Biomed Sci.

[5] Riordan T (2007). Human infection with Fusobacterium necrophorum (Necrobacillosis), with a focus on Lemierre's syndrome. Clin Microbiol Rev.

[6] Poplin V, McKinsey DS (2018). Arcanobacterium brain abscesses, subdural empyema, and bacteremia complicating Epstein-Barr virus mononucleosis. Kans J Med.

[7] Greenlee JE (2003). Subdural empyema. Curr Treat Options Neurol.

[8] Chang CY, Chai CS, Ong EL (2020). Streptococcus sanguis brain abscess as an initial manifestation of pulmonary arteriovenous malformation. Clin Case Rep.

